# Tumour Lysis Syndrome Occurring in a Patient with Metastatic Gastrointestinal Stromal Tumour Treated with Glivec (Imatinib Mesylate, Gleevec, STI571)

**DOI:** 10.1155/2007/82012

**Published:** 2007-11-20

**Authors:** Elizabeth M. Pinder, Gurprit S. S. Atwal, Abraham A. Ayantunde, Sarah Khan, Mike Sokal, Tom McCulloch, Simon L. Parsons

**Affiliations:** ^1^Department of Surgery, Nottingham City Hospital, Hucknall Road, Nottingham NG5 1PB, UK; ^2^Department of Pathology, Nottingham City Hospital, Hucknall Road, Nottingham NG5 1PB, UK; ^3^Department of Oncology, Nottingham City Hospital, Hucknall Road, Nottingham NG5 1PB, UK

## Abstract

Tumour lysis syndrome (TLS) is a rare side effect of chemotherapy for solid tumours. It describes the metabolic derangements following rapid and extensive tumour cell death following a good response to chemotherapy. Symptoms are those of metabolic derangement and renal failure. Treatment involves rehydration and correction of metabolic abnormalities. TLS should be considered in high risk groups. We report a case of TLS in a patient with metastatic gastrointestinal stromal tumour treated with imatinib mesylate. To our knowledge, this is the first reported case.

## 1. INTRODUCTION

Gastrointestinal stromal tumour (GIST) is derived from mesodermal tissue but its pathogenesis remains unclear [1]. It predominantly occurs in the wall of the gastrointestinal tract and most are found in the stomach and small intestine but have also been reported in the colon, rectum, oesophagus, and extra-intestinal sites such as omentum, mesentery, and retroperitoneum [2]. They occur in persons of either sex usually over the age of 40 years [3]. The incidence is estimated to be from 1000 to 6000 new cases a year in the United States [2, 3]. The increasing use of immunohistochemistry, in particular CD117, has increased awareness of existence of GISTs amongst surgical pathologists [3].

Most GISTs have a gain of function mutation in the c-kit proto-oncogene resulting in ligand independent activation of the KIT receptor tyrosine kinase and unopposed stimulus for cell growth. Glivec selectively binds to and inhibits the activity of a small number of tyrosine kinases including ABL, BCR ABL, platelet derived growth factor receptor, and KIT [2, 4]. Results have shown that inhibition of active mutant c-kit tyrosine kinase by glivec is an effective therapy for GISTs [4, 5].

Tumour lysis syndrome (TLS) is commonly seen following the treatment of exquisitely chemosensitive tumours, such as haematological malignancies, and has been reported in various solid tumours [6], but to our knowledge this is the first reported case of TLS occurring in a patient with metastatic GIST following treatment with imatinib.

## 2. CASE REPORT

An 81-year-old man presented in 2002 with a two-day history of melaena and dizziness. He gave no history of peptic ulcer disease, NSAID use, and denied significant alcohol intake. His past medical history included systemic hypertension, noninsulin dependent diabetes mellitus, ischaemic heart disease and gout which were all well controlled on medication including allopurinol. He was also under regular review for chronic lymphocytic leukaemia. This was stable and he was not receiving active treatment.

**Figure 1:**
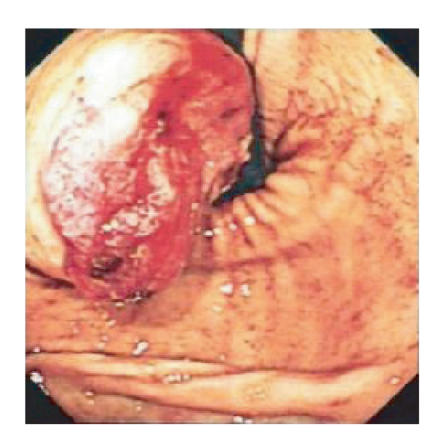
Findings at endoscopy. Note the large ulcerative gastric mass on the greater curve of the stomach.

On examination he was haemodynamically stable and his abdomen was soft and nontender, with no palpable masses. Per rectum, digital examination revealed only tarry black stools consistent with melaena. Haemoglobin on admission was 13 g/dL, urea 28 mmol/L, creatinine 142 mmol/L, liver function tests and clotting were normal. Endoscopy showed a large focally ulcerative gastric mass on the greater curve with prominent underlying vasculature (see Figure 1). The lesion was injected with adrenaline and coagulated with argon plasma resulting in haemostasis. Over the following two days, he had further episodes of melaena associated with a haemoglobin of 6.7 g/dL requiring multiple blood transfusions.

**Figure 2:**
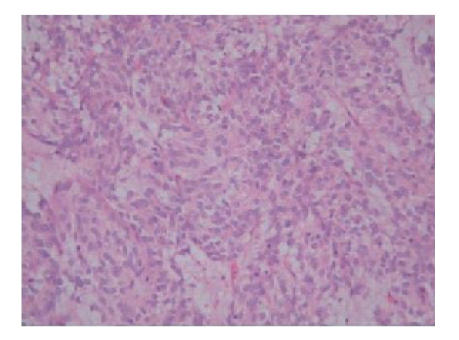
Photomicrograph (x200) of the original resection specimen showing a cellular and epithelioid neoplasm.

Emergency laparotomy revealed a large ulcerated lesion on the anterior wall of the distal half of the stomach not involving the liver. A partial anterior gastrectomy with wide excision of the gastric mass was performed. Histologically, the tumour consisted of epithelioid cells with eosinophilic cytoplasm, prominent nucleoli with a mitotic count of 9 per 30 high-power fields arranged in a whorled and palisaded pattern with intervening myxoid stroma. Immunohistochemistry demonstrated tumour cell positivity for CD 117, smooth muscle actin, and CD 34. Staining for S100 protein, chromogranin and synaptophysin were negative, therefore supporting the morphological diagnosis of GIST (see Figure 2). Surgical excision was complete and he made a good recovery.

**Figure 3:**
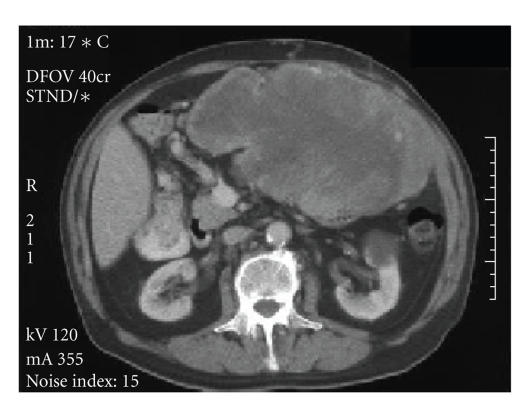
Computerised tomography scan of large abdominal mass measuring 20 × 11 × 25 cm.

Initial postoperative follow up was performed but unfortunately he was lost to follow up. Thirty-two months later, he was readmitted with sudden onset abdominal pain and reduced appetite with no change in bowel habit or weight loss. On examination, there was a large left upper quadrant mass and mild tenderness in the left iliac fossa. Haemoglobin was 11 g/dL, he had mild renal impairment (serum urea 11.3 mmol/L, creatinine 139 mmol/L) and there was an isolated rise in alkaline phosphatase (456 IU/L). Computerised tomography (CT) scan demonstrated a large abdominal mass involving the mesentery and abdominal wall musculature measuring 20 × 11 × 25 cm (see Figure 3). There was no evidence of liver metastases. Ultrasound guided biopsy of the abdominal mass confirmed the diagnosis of recurrent metastatic GIST. Surgery was considered inappropriate and imatinib 400 mg once daily was commenced. Two days later, he became acutely short of breath and oedematous with poor urine output. Imatinib therapy was stopped and his serum biochemistry showed classic features of TLS with renal impairment, hyperuricaemia, elevated lactate dehydrogenase (LDH), hyperkalaemia, and metabolic acidosis (see Table 1). He was transferred to the High Dependency Unit where he received CPAP, 2 units of blood, treatment to correct potassium levels, isoket, and a furosemide intravenous infusion. Further intensive treatment including haemofiltration was considered inappropriate. His urine output initially improved, however the peripherial oedema did not appear to respond to intravenous furosemide infusion. Despite supportive treatment, his condition deteriorated and he died 11 days after receiving his initial dose of imatinib.

Autopsy findings demonstrated a large mottled friable grey tumour mass hanging on the under surface of the left hemidiaphragm extending towards the left iliac fossa, measuring 20 × 15 × 12 cm but no bleeding into the peritoneum. It fragmented easily and showed significant intratumour haemorrhage. Within the tumour were occasional areas of grey firm fleshy tissue. Two separate tumour nodules were present in the omentum and serosa of the distal sigmoid colon, consisting largely of grey firm fleshy tissue measuring 8 and 5 cm, respectively. The main mass had invaded and perforated the colonic splenic flexure.

**Figure 4:**
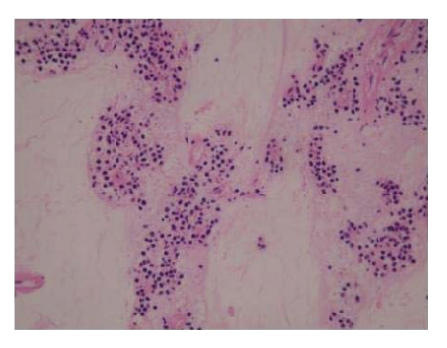
Photomicrograph (x200) of representative area of tumour at post mortem. Note the small pyknotic nuclei and areas of amorphous myxoid stroma representing areas where the tumour cells have disappeared. Other areas were virtually acellular.

**Figure 5:**
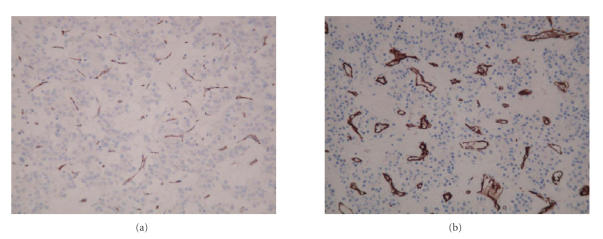
(a) Photomicrograph of representative area of tumour at resection and (b) at post mortem stained with CD31, an endothelial marker

The perforation was localised and walled off with only localised inflammation and no evidence of generalised peritonitis or generalised sepsis elsewhere (e.g., splenic softening). Although the perforation may have contributed to morbidity, it is our opinion, due to its limited extent, that it was not the primary cause of death. Histologically, the vast majority of the main mass was hypocellular consisting of scattered tumour cells staining positive with CD117 with pyknotic nuclei and condensed cytoplasm (resembling mast cells) in an abundant myxoid stroma (see Figure 4). The changes demonstrated dramatic tumour cell kill consistent with the effects of imatinib. Areas of fleshy tumour (including the two separate nodules) were microscopically identified as viable tumour. The vessel density of the tumour was assessed at resection and post mortem, the latter in both in areas of tumour melt and near normality. The vessel density appeared similar, but in the autopsy slides the vessels, although of the same delicate calibre, are significantly more dilated (see Figure 5). This is likely to represent direct effect of the drug and theoretically could indicate leakiness of the vessels. Alternatively but less likely is that it represents normal post mortem autolysis. Nevertheless, the vascular density is the same and thus the tumour “necrosis” cannot be attributed to pure ischaemia.

Post mortem histology showed viable deposits of CLL in the myocardium, liver, spleen, and kidneys and no evidence to suggest direct effect of imatinib. White cell count was 14.30 three days prior to starting imatinib and 14.3 three days after imatinib was commenced. There were no other significant pathologies to cause death, although there was evidence of ischaemic heart disease and a small pumonary embolus. The cause of death was attributed to tumour lysis syndrome occurring as a direct consequence of imatinib treated GIST.

## 3. DISCUSSION

Tumour lysis syndrome is defined as a life threatening metabolic emergency usually associated with the chemotherapeutic treatment of certain malignancies, particularly haematological. Rapid cell death occurs a few hours to days after administration of treatment resulting in the release of intracellular potassium, phosphate, uric acid, and other purine metabolites, which overwhelm the kidneys' capacity for clearance. Hyperkalaemia, hyperphosphataemia, hyperuricaemia, and hypocalcaemia result. Underlying renal impairment potentiates the risk of TLS.

This patient certainly had many of the features of TLS, although he did not require renal replacement therapy as follows.

- There was massive tumour cell kill seen at post mortem examination, hyperkalaemia, hyperuricaemia, and hyperphosphataemia.
- Uric acid was not measured pretreatment, but the patient was on long-term allopurinol for gout, and it is therefore likely that the serum uric acid was normal when starting imatinib. It rose by at least by 40*%* on day 3.
- Incipient renal failure was corrected with vigorous fluids. However, hyperkalaemia remained a problem. The potassium, which was at the upper end of the normal range for over a month preceding treatment, rose considerably.

The significant side effects of imatinib that could have been implicated in his death did not occur—at post mortem there was neither ascites that might have caused respiratory embarrassment, nor intraperitoneal bleeding from the very haemorrhagic tumours.

At post mortem examination, there was no significant intra-abdominal sepsis, only a localised peritonitic reaction around the splenic flexure. Other organs showed the expected changes for a man of eighty one years old, that is, moderate coronary artery disease, atheroma of the thoracic and abdominal aorta, and congested lungs.

Although comorbid disease may have contributed to his death, by far the greatest element was the tumour lysis induced by imatinib. Manoeuvres to prevent this such as starting with a lower dose of imatinib are thought not to be appropriate for fear of not bringing the disease under control (J. Verweij, personal communication).

### 3.1. Literature review

Primary surgical excision with good margins is the mainstay of treatment of GIST with an overall five-year survival rate for localised disease of 54*%* [7]. The response rate with cytotoxic chemotherapy is extremely poor [3–5]. The risk of recurrence is high with the liver and peritoneal surfaces most commonly affected [3, 8]. Imatinib was first utilised for metastatic GIST in 2000 [5] and has been consistently shown to be the most specific and effective treatment to date [2–5]. In general, adverse events are minimal and tend to include mild fatigue, periorbital oedema, nausea, diarrhoea, intermittent muscle cramping, rash, headache, and abdominal pain [2, 4]. In approximately 5*%* of cases, severe toxicity grade 3 to 4 adverse events occur including gastrointestinal or intratumour haemorrhage due to massive tumour necrosis, which rarely can be fatal [3]. This is the first reported case of TLS occurring in a patient with metastatic GIST treated with imatinib.

Imatinib is also an effective treatment for chronic myeloid leukaemia and a case report has been published of TLS occurring in one patient [9].

The most reliable parameters for TLS are laboratory indices and clinical signs and symptoms, which typically occur 12–72 hours after commencement of cytotoxic therapy [10].

Although nonhaematological malignancies are considered low risk for developing TLS due to longer doubling time, low growth fraction, and slow response to treatment compared with lymphoproliferative malignancies, there are numerous case reports in patients with different tumours including small cell lung cancer, medulloblastoma but none in patients with GIST. Such solid tumours tend to be very chemosensitive, although TLS can occur in less sensitive tumours if bulky metastatic disease is present.

Risk factors for TLS include bulky disease, elevated pretreatment LDH, compromised renal function, potentially nephrotoxic drugs, and raised uric acid levels. The patient discussed here had several risk factors: dehydration secondary to diarrhoea, compromised renal function, bulky tumour, and use of nephrotoxic drugs (metformin, spironolactone).

Much of the literature on TLS stresses the importance of prevention by having a high index of suspicion for patients with large chemosenstitive tumours. This can be achieved by prophylactic treatment with allopurinol, pretreatment hydration, and alkalinization of urine [11]. There are limitations in the use of allopurinol including slow onset of action, insufficient efficacy in many high-risk patients (57*%* in hyperuricamic patients) [12] and the possibility of allergic reactions and interactions with chemotherapeutic agents. There is also evidence to suggest that allopurinol is no longer the recommended drug for prophylaxis or treatment of TLS due to its inability to lower already established uric acid levels. It also can lead to Xanthine accumulation which can crystallize and precipitate in the renal tubules [13]. An alternative to allopurinol is rasburicase, a recombinant urate oxidase which has been shown to be more effective than allopurinol in preventing and treating hyperuricaemia [14]. This drug is not without side effects or cost and is used mainly in haematological malignancies with little experience in solid tumours. It will however reduce the incidence and severity of TLS.

We thus advise caution when treating advanced GIST with imatinib and suggest a full assessment of risk factors for TLS which include high tumour burden (high white cell count *>*50 × 10^9^/L and/or high LDH levels), elevated uric acid levels, intensive cytoreductive therapy, and poor hydration [10].

## Figures and Tables

**Table 1:** Tabulation of renal function over a 47-day period starting from 35 days prior to treatment with imatinib to 11 days post treatment. LDH = lactate dehydrogenase. Normal ranges: haemoglobin (13.0–18.0 g/dL), white cell count (4.0–11.5E9/l), sodium (135–145 mmol/L), potassium (3.5–5.3 mmol/L), urea (1–6.5 mmol/L), creatinine (60–120 *μ*mol/L), phosphate (0.8–1.4 mmol/L), corrected calcium (2.2–2.6 mmol/L), LDH (230–460 iu/l), uric acid (100–400 *μ*mol/L).

Results	Admission Day *−*35	Day *−*5	Day *−*3	Day +2	Day +3	Day +4	Day +6	Day +10	Day +11 (death)

Haemoglobin	10.9	10.5	9.8	9.6	9.6	9.2	12.6	11.5	11.4
White cell count	10.1	15.10	14.3	14.1	14.3	11.30	8.7	10.9	3.5
Sodium	135	136	136	144	146	150	142	145	146
Potassium	5.1	5.2	5.2	6.3	6.4	4.4	5.4	3.9	4.1
Urea	11.3	19.6	34.8	28	31.4	31.5	17.1	16.5	21.9
Creatinine	139	228	270	148	163	176	115	98	128
Phosphate	—	—	—	—	1.53	—	—	1.15	1.61
Calcium	—	—	—	—	2.43	—	—	2.32	2.31
LDH	—	—	—	—	686	—	—	—	—
Uric acid	—	—	—	—	574	—	—	—	—
